# Paralysie congénitale du IV révélée par une diplopie post chirurgie de la cataracte

**DOI:** 10.11604/pamj.2014.18.329.4853

**Published:** 2014-08-25

**Authors:** Aziz El Ouafi, Med Elmellaoui, Abdelkader Lakataoui

**Affiliations:** 1 Service d'ophtalmologie, Hôpital Militaire Moulay Ismail, Mekhnès, Maroc

**Keywords:** Diplopie, paralysie du IV, cataracte, Diplopia, Fourth nerve palsy, cataract

## Abstract

Les causes de diplopie après une chirurgie de cataracte sont nombreuses. La paralysie congénitale du IV est peu fréquente et diagnostic difficile car elle peut rester longtemps compensée. Nous rapportons un cas qui souligne l'importance de penser, devant une diplopie, à une étiologie congénitale même à un âge avancé.

## Introduction

Les causes de diplopie après une chirurgie de cataracte sont diverses. Une complication chirurgicale est souvent recherchée: luxation d'implant, erreur biométrique, iridectomie mal faite, astigmatisme iatrogène.

## Patient et observation

**Figure 1:**
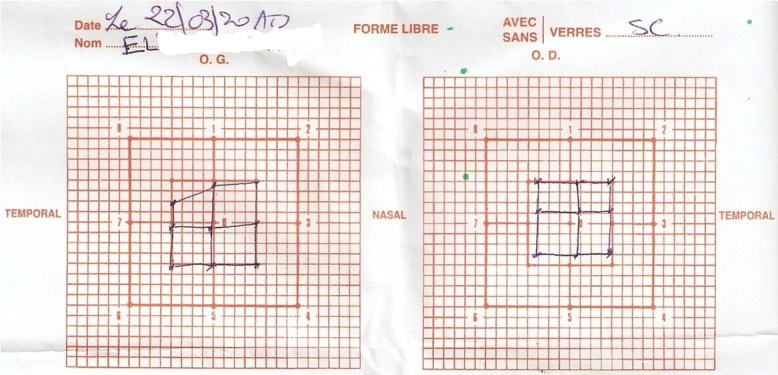
Lancaster montrant la paralysie du IV (atteinte du grand oblique droit, avec hyperaction du droit inferieur gauche et limitation du muscle droit supérieur)

Nous présentons une observation clinique d'un patient âgé de 65 ans, sans antécédents pathologiques, opéré de cataracte sénile aux deux yeux. 6 mois après la chirurgie du 2^ème^ oeil, le patient a présenté une diplopie verticale. Le bilan étiologique a été basé sur l'examen clinique, le Lancaster, le bilan orthoptique et l'IRM. La diplopie est verticale avec gène à la descente des escaliers et à la lecture. Le signe de bielshowsky est positif. Le Lancaster a montré une atteinte du grand oblique droit, avec hyperaction du droit inferieur gauche et limitation du muscle droit supérieur (Figure 1). Le bilan orthoptique a révélé un pouvoir fusionnel à 8 dioptries de loin et 12 dioptries de près. L IRM est normale.

## Discussion

La paralysie congénitale du IV est peu fréquente et diagnostic difficile car elle peut rester longtemps compensée. [1] La décompensation à l’âge de la presbytie est liée à la diminution du pouvoir accommodatif. [2] L'implantation après chirurgie de la cataracte (PMMA) supprime l'accommodation et démasque la pathologie.

## Conclusion

A travers ce cas, les auteurs rappellent la paralysie congénitale du IV comme une cause à évoquer en cas de diplopie postopératoire. [3] L'examen clinique, le Lancaster, et le bilan orthoptique sont les éléments clés du diagnostic.
